# The effects of probiotics supplementation on metabolic health in pregnant women: An evidence based meta-analysis

**DOI:** 10.1371/journal.pone.0197771

**Published:** 2018-05-21

**Authors:** Jia Zheng, Qianyun Feng, Sheng Zheng, Xinhua Xiao

**Affiliations:** 1 Department of Endocrinology, Key Laboratory of Endocrinology, Ministry of Health, Peking Union Medical College Hospital, Chinese Academy of Medical Sciences & Peking Union Medical College, Beijing, China; 2 Tianjin University of Traditional Chinese Medicine, Tianjin, China; 3 Department of Pediatrics, The Second Teaching Hospital of Tianjin University of Traditional Chinese Medicine, Tianjin, China; University of Missouri Columbia, UNITED STATES

## Abstract

The prevalence of maternal obesity and gestational diabetes mellitus (GDM) is increasing rapidly. Probiotics supplementation have been shown to improve metabolic health in humans. In our study, we aimed to evaluate the effects of probiotics supplementation on metabolic health and pregnancy complications in pregnant women. The literature search, data extraction and quality assessment were performed, and data were synthesized in accordance with standardized guidelines. Ten randomized clinical trials with eligible data were included in our meta-analysis. For pregnant women with GDM, we found negative correlations between probiotics supplementation and fasting serum insulin (OR -2.94, 95%CI [-5.69, -0.20], p = 0.04) and homoeostasis model assessment for insulin resistance (HOMA-IR) (OR -0.65, 95%CI [-1.18, -0.11], p = 0.02). There were no significant correlations between probiotics supplementation and lipid levels in women with GDM, including total cholesterol (OR -2.72, 95%CI [-17.18, 11.74], P = 0.71), high density lipoprotein cholesterol (HDL-c) (OR -0.29, 95%CI [-3.60, 3.03], P = 0.87), low density lipoprotein cholesterol (LDL-c) (OR -0.38, 95%CI [-18.54, 17.79], P = 0.97), or triglycerides (OR -12.83, 95%CI [-36.63, 10.97], P = 0.29). For healthy pregnant women, probiotics supplementation were negatively associated with fasting serum insulin (OR -3.76, 95%CI [-4.29, -3.23], P < 0.00001) and HOMA-IR (OR -0.57, 95%CI [-1.08, -0.06], p = 0.03). However, no significant correlations were observed between probiotics supplementation and fasting plasma glucose (FPG) (OR -2.02, 95%CI [-5.56, 1.52], p = 0.26). Thus, our study revealed that probiotics supplementation during pregnancy have beneficial effects on glucose metabolism, rather than lipid metabolism among pregnant women.

## Introduction

The prevalence of obesity and type 2 diabetes mellitus (T2DM) in adulthood is increasing dramatically [1]. However, the pathogenesis of diabetes mellitus has not been fully demonstrated. Increasing evidence shows that maternal environment, especially metabolic status during pregnancy, is a critical element determining the development of metabolic diseases, such as obesity and T2DM in offspring [2–4]. It is estimated that one in six births is affected by gestational diabetes mellitus (GDM) in 2017 [1]. About 50% women with childbearing ages and 20–25% pregnant women in Europe were overweight and obese [5]. Obese pregnant women are at higher risks of developing GDM, that can affect metabolic health of mothers and newborns.

Obese pregnant women and women with GDM are associated with higher risks of maternal and fetal morbidity and mortality [6]. For the mothers, it can increase the susceptibility of undergoing a caesarian section, preeclampsia and the development of T2DM postpartum [6–8]. For their fetuses, it increases the risks of macrosomia, polyhydramnios, shoulder dystocia, preterm birth and neonatal hypoglycemia [6–8]. Offspring of women with GDM may be more likely to develop obesity, insulin resistance and T2DM in adulthood [9]. In addition, maternal obesity is associated with neonatal adiposity and obesity in childhood. The programming effect was known as “Developmental Origins of Health and Disease (DOHaD)” hypothesis, and it has now been widely accepted [10, 11]. Therefore, obese pregnant women and women with GDM not only increase the incidecne of adverse pregnancy outcomes, but also have long-term effects on metabolic health of mothers and their offspring.

Lifestyle interventions, including diet and exercise have been demonstrated to prevent against maternal obesity and GDM. However, it has proven to be challenging, with low compliance and limited efficacy [12]. In recent years, gut microbiota is shown to be associated with obesity and several metabolic diseases [13]. It is related with developmental biology, including both prenatal and postnatal growth [14]. Probiotics supplementation have been shown to regulate microbiota dysbiosis, and probiotics are emerging as an effective intervention to improve whole body health in humans, and even in term and preterm infants [15, 16]. However, its roles in regulating metabolic health during pregnancy remain unclear, especially in obese pregnant women and women with GDM. Therefore, our study was aimed to determine the effects of probiotics supplementation on metabolic health in pregnant women, including healthy pregnant women, obese pregnant women and women with GDM.

## Materials and methods

### PRISMA guideline and PICO principle

This systematic review has been registered in PROSPERO titled as “The effects of probiotics supplementation on glucose metabolic health in pregnancy women” (register number: CRD42017060312). The review was conducted and reported in accordance with Preferred Reporting Items for Systematic Reviews and Meta-Analysis (PRISMA) guideline [17] and PICO principle, including P-Population: pregnant women, including healthy pregnant women, obese pregnant women and women with GDM; I-Intervention: probiotics supplementation; C-Comparison: metabolic health involving probiotics supplementation as an intervention during pregnancy, as compared with placebo; O-Outcome: changes in metabolic parameters and pregnancy outcomes with probiotics supplementation.

All the literature search, data extraction and quality assessment were performed independently and in duplicate by two authors (QYF and SZ) using a standardized approach. Any disagreements were documented and resolved by discussion between data collectors along with the principal investigator (JZ and XHX).

### Data sources, search strategy, and selection criteria

Pubmed, Embase, Cochrane Library and Clinicaltrials.gov databases were systematically screened for relevant studies. All literatures were published updated to February 2017 and the language was limited to English. The main search terms were a combination of MESH terms and text words for probiotics, pregnancy and metabolic parameters, with the following terms: “probiotics” OR “bacteria” AND “pregnancy” OR “gestation” OR “parturition” AND “glucose” OR “insulin” OR “HbA1c” OR “glycosylated hemoglobin A1c” OR “glycemic control” OR “metabolism”. All the articles, including conference abstracts were reviewed. All the literatures were reviewed and additional relevant references quoted in searched articles were also screened. All the literatures were managed by Endnote X7 software.

All studies about changes in metabolic parameters before and after probiotics supplementation in pregnant women were screened. In our study, only randomized clinical trials (RCTs) were included, since RCT provides relatively strong evidence for the efficacy of clinical trials [18]. The studies that met the following criteria were included: (1) studies about the effects of probiotics on pregnancy outcomes; (2) measured glucose and lipid metabolism parameters; (3) sufficient data for evaluation. Studies were excluded based on the following criteria: (1) studies in which clinical outcomes of pregnancy could not be ascertained; (2) observational studies; (3) preclinical studies; (4) reviews or conference abstracts or case reports or editorials or book chapters.

### Data extraction

Two authors (JZ and QYF) independently screened the title and abstract of each article for the relevance of subjects, quality of clinical trial and eligibility for inclusion. The following information was collected from each included study using a standardized protocol and reporting form: first author, year of publication, country, study design, subjects, probiotic interventions (including probiotics species, probiotics counts measured by colony-forming unit (cfu)), intervention duration, sample size, mean age, primary outcome and secondary outcomes that passed the two rounds of screening. The detailed information was shown in Table 1.

**Table 1:** Characteristics of the studies included in the systematic review.

Study ID	Subjects	Year	Country	Study Design	Probiotic species and counts	Intervention Duration	Sample size	Mean age	Primary outcome	Secondary outcomes
Laitinen et al. [23]	Healthy pregnant women	2009	Finland	RCT	*Lactobacillus rhamnosus* GG (10^10^ cfu) and *Bifidobacterium lactis* Bb12 (10^10^ cfu)	First trimester until end of exclusive breast-feeding	256	30 years	FPG, HbA1c, insulin and HOMA and QUICKI indices	Dietary energy-yielding nutrients
Luoto et al. [24]	Healthy pregnant women	2010	Finland	RCT	*Lactobacillus rhamnosus* GG (10^10^ cfu) and *Bifidobacterium lactis* Bb12 (10^10^ cfu)	First trimester until end of exclusive breast-feeding	256	30 years	Maternal glucose metabolism, incidence of GDM, adverse pregnancy outcomes	The duration of exclusive and total breastfeeding
Ilmonen et al. [25]	Healthy pregnant women	2011	Finland	RCT	*Lactobacillus rhamnosus* GG (10^10^ cfu) and *Bifidobacterium lactis* Bb12 (10^10^ cfu)	First trimester until end of exclusive breast-feeding	256	30 years	The risk of central adiposity	The intakes of foods and nutrients during pregnancy
Asemi et al. [26]	Healthy pregnant women	2013	Iran	RCT	*Lactobacillus acidophilus* LA5 and *Bifidobacterium animalis* Bb12 (10^7^ cfu)	Start at the third trimester for 9 weeks	70	18–30 years	Plasma glucose levels, insulin and HOMA	BMI changes
Jamilian et al. [27]	Healthy pregnant women	2016	Iran	RCT	three probiotic spices *Lactobacillusacidophilus*, *Lactobacillus casei*, *Bifidobacterium bifidum* (2×10^9^ cfu)	Start from 9 weeks of gestation for a duration of 12 weeks	60	18–37 years	Assessment of anthropometric measures	Fasting glucose, insulin, HOMA-IR, HOMA-β, QUICKI, serum lipids
Lindsay et al. [28]	Pregnant women with a BMI (30.0–39.9)	2014	Ireland	RCT	*Lactobacillus salivarius* UCC118 (10^9^ cfu)	From 24 to 28 week of gestation	175	31 years	Maternal glucose metabolism, incidence of GDM, adverse pregnancy outcomes	NA
Dolatkhah et al. [29]	Pregnant women with GDM	2015	Turkey	RCT	Four bacterial strains (Lactobacillus acidophilus LA-5, Bifidobacterium BB-12, Streptococcus thermophilus STY-31 and Lactoba- cillus delbrueckii bulgaricus LBY-27) (> 4 × 10^9^ cfu)	From diagnosis until delivery for 8 weeks	64	18–45 years	Weight gain, fasting blood glucose, insulin, HOMA-IR, QUICKI	NA
Lindsay et al. [30]	Pregnant women with GDM	2015	Ireland	RCT	*Lactobacillus salivarius* UCC118 (10^9^ cfu)	From diagnosis until delivery for 6 weeks	149	33 years	Post-intervention maternal fasting glucose, metabolic parameters, gestational weight gain	Pharmacological therapy and neonatal birth weight
Karamali et al. [31]	Pregnant women with GDM	2016	Iran	RCT	Three probiotic species *Lactobacillusacidophilus*, *Lactobacillus casei*, *Bifidobacterium bifidum* (2 × 10^9^ cfu)	From diagnosis until delivery for 6 weeks	60	18–40 years	FPG, insulin, HOMA-IR, HOMA-β, QUICKI	Lipid concentrations
Jafarnejad et al. [32]	Pregnant women with GDM	2016	Iran	RCT	VSL#3 probiotic capsule with 112.5 × 10^9^ cfu/capsule of eight strains of lactic acid bacteria	From diagnosis until delivery for 8 weeks	82	32 years	FPG, HbA1c, HOMA-IR, and insulin levels	NA

RCT, randomized controlled trial; GDM, gestational diabetes mellitus; cfu, colony-forming unit; FPG, fasting plasma glucose; HbA1c, glycosylated hemoglobin A1c; HOMA, homoeostasis model assessment; HOMA-IR, HOMA for insulin resistance; HOMA-β, HOMA for β-cell function; QUICKI, quantitative insulin sensitivity check index; NA, not available.

### Assessment of study quality

Two authors (QYF and SZ) independently screened the included articles and assessed the study quality [19]. Ten criteria are generally used to assess the sufficiency of reporting, including: (1) Was the randomization method appropriate? (2) Was the allocation sequence concealed? (3) Were the participants blind to the intervention? (4) Were the outcome assessors blind to the intervention? (5) Was the outcome measurement performed in the same manner? (6) Were similarly trained individuals administering the intervention across groups? (7) Were all the withdrawals described? (8) Were all originally randomized participants analyzed in the groups they were assigned to? (9) Was clustering at the group level accounted for in the analyses? (10) Were the groups similar at baseline?

For each of these criteria, it indicated as a “Yes” judged as fulfilling the criterion, or indicated as a “No” for not fulfilling it, or indicated as “Not Reported (NR)” due to insufficient information. If the article has included a complete description regarding the process and outcome of each criterion, it can be designated as “Yes”. If the investigators would be unable to replicate the process based on unclear information, due to insufficient information, it was designated as a “NR” for that criterion. A complete lack of reporting or an erroneous method was marked as “No”. The evaluation of risk-of-bias criteria for each study was shown in Table 2.

**Table 2:** Quality assessment of all the included studies in the systematic review.

Study ID	Laitinen et al.[23]	Luoto et al.[24]	Ilmonen et al.[25]	Asemi et al.[26]	Jamilian et al.[27]	Lindsay et al.[28]	Dolatkhah et al. [29]	Lindsay et al.[30]	Karamali et al. [31]	Jafarnejad et al. [32]
**1. Was the randomization method to groups appropriate?**	Yes	Yes	Yes	Yes	Yes	Yes	Yes	Yes	Yes	Yes
**2. Was the allocation sequence concealed from those assigning patients to groups?**	Yes	Yes	Yes	Yes	Yes	Yes	Yes	Yes	Yes	Yes
**3. Were the participants blind to the intervention?**	Yes	Yes	Yes	Yes	Yes	Yes	Yes	Yes	Yes	Yes
**4. Were the outcome assessors blind to the intervention?**	Yes	Yes	Yes	No	Yes	Yes	Yes	Yes	Yes	Yes
**5. Was the outcome measurement performed in the same manner?**	Yes	Yes	Yes	Yes	Yes	NR	Yes	NR	Yes	NR
**6. Were similarly trained individuals administering the intervention across groups?**	NR	NR	NR	NR	Yes	Yes	NR	Yes	NR	Yes
**7. Were all the withdrawals described?**	Yes	Yes	Yes	Yes	Yes	Yes	Yes	Yes	Yes	Yes
**8. Were all originally randomized participants analyzed in the groups they were assigned to?**	Yes	Yes	Yes	NR	NR	Yes	Yes	Yes	Yes	Yes
**9. Was clustering at the group level accounted for in the analyses?**	NR	NR	NR	Yes	Yes	NR	Yes	Yes	Yes	Yes
**10. Were the groups similar at baseline?**	Yes	Yes	Yes	Yes	Yes	Yes	Yes	Yes	Yes	Yes

NR, Not Reported.

## Statistical analysis

RevMan 5.3 software was used for this meta-analysis. The Cochran's *Q* test, and *I*^2^ test were all performed to judge the heterogeneity among the studies. Heterogeneity was also considered to be significant at P<0.1 for the Q statistic. I^2^ values of 25%, 50% and 75% corresponded to low, moderate and high levels of heterogeneity, respectively [20]. The selection of fixed-effects model or random-effects model was depended on the size of the heterogeneity among the included studies [21]. Sensitivity analysis was performed by successively excluding the low quality studies to assess the stability of the outcomes [22]. Pooled odds ratios (ORs) were reported with 95% confidence intervals (CIs), and a two-tailed P < 0.05 was considered statistically significant for all analyses.

## Results

### Studies included and participant characteristics

**Fig 1:**
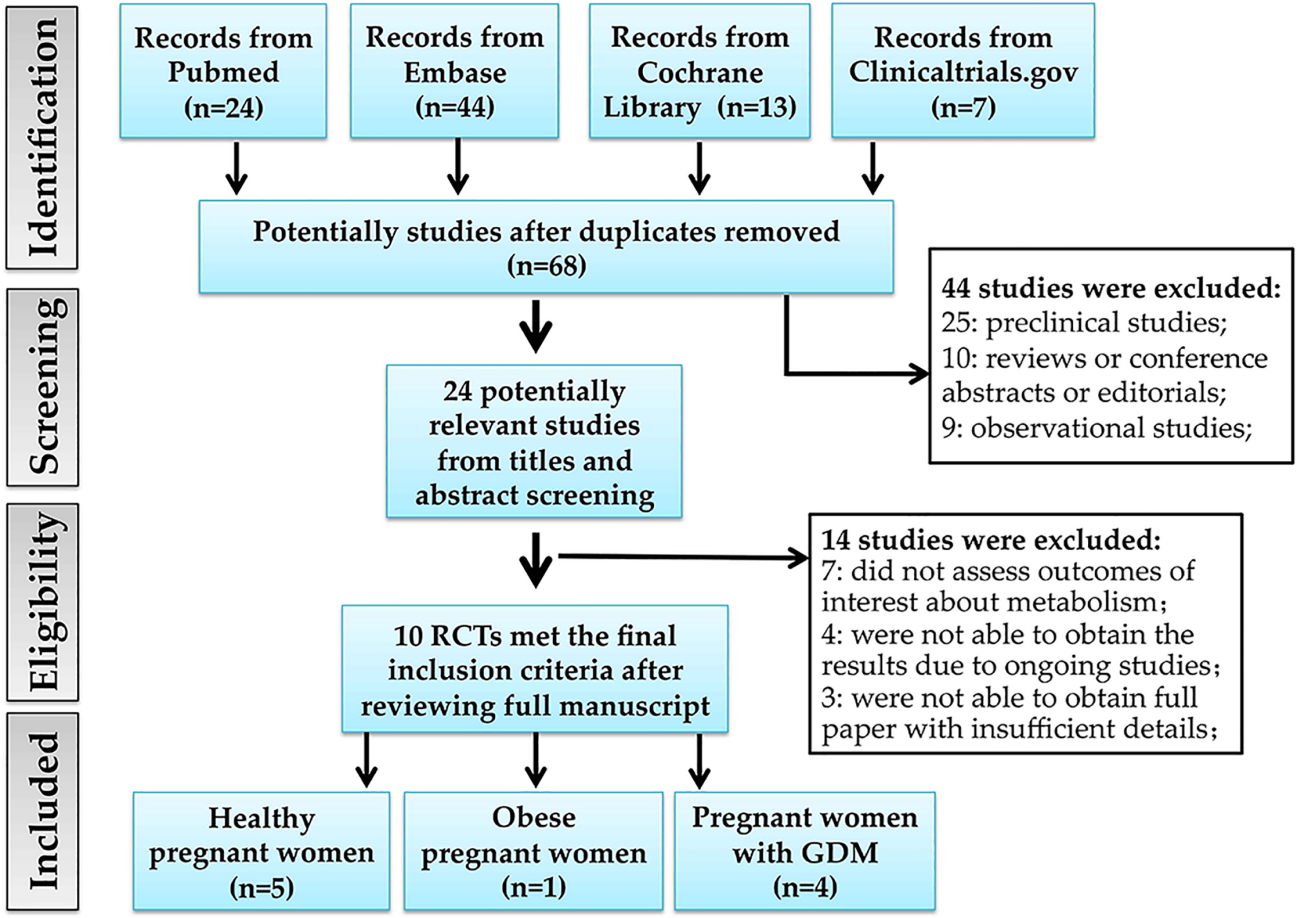
Flow diagram of literature search and included studies. RCT, randomized controlled trial; GDM, gestational diabetes mellitus.

Fig 1 summarizes the selection process of eligible studies. We identified 88 potentially eligible literatures and 68 were kept after removing duplicates. 44 articles were excluded due to preclinical studies, reviews or conference abstracts or case reports or editorials or book chapters or observational studies. 24 potential studies were further reviewed after reading the full article. Next, 14 articles were excluded based on the outcomes of interest, ongoing clinical trials and inaccessible for full paper. Finally, 10 RCTs with eligible data were included in the systematic review [23–32]. Of the ten studies, three studies were from the same study cohort [23–25], and two studies were from another study cohort [27, 31]. Of the ten RCTs, five studies were aimed to evaluate probiotics and its effects on metabolic health in healthy pregnant women [23–27]. Only one study was about the effect of probiotics on metabolic health in obese pregnant women [28]. The remaining four studies aimed to assess the effect of probiotics supplementation on metabolic health in women with GDM [29–32]. The enrollment sample size ranged from 60 to 256 subjects. The detailed characteristics of the studies are shown in Table 1, and the outcomes of each study are shown in Table 3.

**Table 3:** The outcomes of studies included in the systematic review.

	Maternal outcomes		Fetal outcomes	
Study ID	Positive outcomes	Negative outcomes	Positive outcomes	Negative outcomes
Laitinen et al. [23]	Reduced plasma glucose (P = 0.025) and improved insulin sensitivity (P = 0.028) in diet/probiotic group, during pregnancy and post-partum;	NA	NA	NA
Luoto et al. [24]	Reduced GDM frequency in diet/probiotic group (13%) compared to diet/placebo (36%) and control/placebo (34%) groups (P = 0.03);	NA	Diminished the risk of larger birth size: birth weight (P = 0.035) and birth length (P = 0.028);	No significant differences in prenatal or postnatal growth rates;
Ilmonen et al. [25]	Lowered central adiposity;	NA	NA	NA
Asemi et al. [26]	Significant insulin levels and HOMA-IR changes;	No difference of serum insulin levels and HOMA-IR score between group;	NA	NA
Jamilian et al. [27]	Decreased serum insulin concentrations, HOMA-IR, HOMA-β and serum triglycerides levels; Increased QUICKI;	NA	NA	NA
Lindsay et al. [28]	NA	No differences in the incidence of impaired glycemia, birth weight, or pregnancy outcomes;	NA	No differences of number of large-for-gestational-age babies, or admission to the NICU or gestational age at delivery or Apgar score;
Dolatkhah et al. [29]	Lower weight gain, decreased fasting blood glucose and reduction of insulin resistance index;	No difference of insulin sensitivity index;	NA	NA
Lindsay et al. [30]	Lower total and LDL cholesterol;	No differences in post-intervention fasting glucose, requirement for pharmacological therapy or birth weight;	NA	No difference of neonatal anthropometry, assessed by absolute birthweight, birthweight centile, small or large for gestational age, macrosomia, head circumference or length;
Karamali et al. [31]	Decreased fasting plasma glucose, serum insulin levels, HOMA-IR and HOMA-β, increase in QUICK; decreased serum triglycerides and VLDL concentrations;	No significant changes in other lipid profiles;	NA	No difference of the newborns’ mean weight, height, head circumference, caesarean section rate or birth of macrosomic infants;
Jafarnejad et al. [32]	Significant differences in insulin levels and HOMA-IR;	FPG, HbA1c, HOMA-IR, and insulin levels remained unchanged;	NA	NA

GDM, gestational diabetes mellitus; HOMA, homoeostasis model assessment; HOMA-IR, HOMA for insulin resistance; HOMA-β, HOMA for β-cell function; QUICKI, quantitative insulin sensitivity check index; VLDL, very low-density lipoprotein; NICU, neonatal intensive care unit; NA, not available

### Probiotics and its effectiveness in women with GDM

Four studies were included to assess the effect of probiotics supplementation on metabolic health in women with GDM [29–32]. For glucose metabolism, comprehensive integration and analyses revealed negative correlations between probiotics supplementation and fasting serum insulin (OR -2.94, 95%CI [-5.69, -0.20], p = 0.04) and homoeostasis model assessment for insulin resistance (HOMA-IR) (OR -0.65, 95%CI [-1.18, -0.11], p = 0.02). However, no significant correlations were observed between probiotics supplementation and fasting plasma glucose (FPG) (OR -3.24, 95%CI [-8.77, 2.30], p = 0.25), and quantitative insulin sensitivity check index (QUICKI) index (OR 0.01, 95%CI [0.00, 0.02], p = 0.05) (Table 4). Among the four studies, two studies assessed the effects of probiotics supplementation on lipid metabolism in women with GDM. However, there were no significant correlations between probiotics supplementation and lipid levels in women with GDM, including total cholesterol (OR -2.72, 95%CI [-17.18, 11.74], P = 0.71), high density lipoprotein cholesterol (HDL-c) (OR -0.29, 95%CI [-3.60, 3.03], P = 0.87), low density lipoprotein cholesterol (LDL-c) (OR -0.38, 95%CI [-18.54, 17.79], P = 0.97), or triglycerides (OR -12.83, 95%CI [-36.63, 10.97], P = 0.29) (Table 4). Among the four studies, two studies evaluated the effects of maternal probiotics supplementation on pregnancy outcomes. Lindsay et al. showed that there was no difference in neonatal anthropometry, assessed by absolute birth weight, birth weight centile, small or large for gestational age, macrosomia, head circumference or length [30]. Karamali et al. also found no difference in mean weight, height, head circumference, caesarean section rate or fetal macrosomia in newborns [31]. Overall, these results demonstrated that probiotic supplementations had beneficial effects on glycemic control, rather than lipid metabolism in women with GDM.

**Table 4:** Pooled-analysis results of probiotics and its effects on metabolic health in pregnant women.

Outcomes	References	Patients	OR/WMD (95% CI)	P value	*I*^*2*^, %	Heterogeneity (P value)
**Gestational diabetes mellitus (GDM)**						
Fasting plasma glucose	[29–32]	288	-3.24 [-8.77, 2.30]	P = 0.25	99%	P < 0.00001
Fasting serum insulin	[29–32]	288	-2.94 [-5.69, -0.20]	P = 0.04	84%	P = 0.0002
HOMA-IR	[29–32]	288	-0.65 [-1.18, -0.11]	P = 0.02	76%	P = 0.006
QUICKI index	[29, 31]	116	0.01 [0.00, 0.02]	P = 0.05	86%	P = 0.008
Total cholesterol	[30, 31]	160	-2.72 [-17.18, 11.74]	P = 0.71	82%	P = 0.02
HDL cholesterol	[30, 31]	160	-0.29 [-3.60, 3.03]	P = 0.87	71%	P = 0.06
LDL cholesterol	[30, 31]	160	-0.38 [-18.54, 17.79]	P = 0.97	92%	P = 0.0005
Triglycerides	[30, 31]	160	-12.83 [-36.63, 10.97]	P = 0.29	74%	P = 0.05
**Healthy pregnant women**						
Fasting plasma glucose	[25–27]	265	-2.02 [-5.56, 1.52]	P = 0.26	92%	P < 0.00001
Fasting serum insulin	[26, 27]	130	-3.76 [-4.29, -3.23]	P < 0.00001	0%	P = 0.45
HOMA-IR	[25–27]	219	-0.57 [-1.08, -0.06]	P = 0.03	86%	P = 0.0009

HOMA-IR, homoeostasis model assessment for insulin resistance; QUICKI, quantitative insulin sensitivity check index; LDL, low-density lipoprotein; HDL, high-density lipoprotein.

### Probiotics and its effectiveness in healthy pregnant women

There are five RCTs about probiotics supplementation in healthy pregnant women and one research group had three publications on various metabolic endpoints of mothers. For glucose metabolism, comprehensive integration and analyses revealed significant correlations between probiotics supplementation and lower fasting serum insulin (OR -3.76, 95%CI [-4.29, -3.23], P < 0.00001) and HOMA-IR (OR -0.57, 95%CI [-1.08, -0.06], p = 0.03). However, no significant correlations were observed between probiotics supplementation and FPG (OR -2.02, 95%CI [-5.56, 1.52], p = 0.26) (Table 4). Only one study examined the infant health outcomes and found that there were no significant differences in prenatal or postnatal growth rates. This study supported the evidence that probiotic supplementation during pregnancy had beneficial effects on glucose metabolism in healthy pregnant women.

### Probiotics and its effectiveness in obese pregnant women

One study was included to investigate the effect of a probiotics supplementation on metabolic health in obese pregnant women [28]. A total of 138 women were recruited in the study and randomly assigned to receive either a daily probiotic (Lactobacillus salivarius UCC118 (10^9^ cfu)) or a placebo capsule from 24 to 28 weeks of gestation in addition to routine antenatal care. The primary outcomes were maternal FPG concentration changes, with the incidence of impaired glycemia including IGT or GDM as a secondary outcome. There were no differences in the incidence of impaired glycemia, birth weight, or pregnancy outcomes [28]. For infant health outcomes, there were no differences in number of large-for-gestational-age babies, admission to the neonatal intensive care unit, gestational age at delivery or Apgar score. The results can be explained by that there were only 15 cases of IGT and 6 cases of GDM among the participants, limiting the statistical power of the study.

## Discussion

With the increasing prevalence of maternal obesity and GDM, it places enormous burden on individual and public health. Preventing obesity, insulin resistance and hyperglycemia during pregnancy shows pronounced benefits for metabolic health [33]. Currently, lifestyle interventions, including diet and exercise, have been demonstrated to prevent and treat GDM [34]. However, compliance with these interventions is low and the efficacy is limited. It showed that a behavioral intervention (including diet and physical activity) in obese pregnant women was not adequate to prevent the incidence of GDM, or to reduce the incidence of large-for-gestational-age infants [35]. Han et al. reviewed 19 trials with 1398 women with GDM and showed that no clear differences were observed among different types of dietary advice for women with GDM [36]. Other management practices, such as medication therapy for GDM are expensive but also do not always reduce the incidence of GDM [12].

According to the World Health Organization, probiotics can confer health benefits on the host microorganisms. Therefore, our systematic review aimed to assess the effects of probiotics supplementation on metabolic health in the pregnant women, including healthy pregnant women, obese pregnant women and women with GDM. Ten RCTs were included in the final systematic review. Five studies [23–27] were aimed to evaluate probiotics and its effects on metabolic health in healthy pregnant women. It demonstrated that probiotic supplementation during pregnancy had beneficial effects on glucose metabolism. One study [28] was about probiotics and metabolic health in obese pregnant women. However, there were no differences in metabolic variables or pregnancy outcomes in obese pregnant women, that may be due to the limited number of relevant studies. The remaining four studies [29–32] aimed to assess the effect of probiotics supplementation on metabolic health in pregnant women with GDM, and showed that probiotic supplementations had beneficial effects on glycemic control, rather than lipid metabolism among women with GDM. Taylor et al. reviewed the effects of probiotics on metabolic outcomes in pregnant women with GDM. Consistently, it also showed that improved glucose metabolism with a significant reduction in HOMA-IR was observed following probiotic supplementation [37]. Taken all together, probiotics supplementation during pregnancy have beneficial effects on metabolic health among pregnant women, including women with GDM, and even healthy pregnant women. Among the included ten studies, only four studies have evaluated the effects of probiotics supplementation on birth weight. However, the sample sizes were relatively small. Thus, the effects of probiotics supplementation on infant health outcomes are uncertain. More studies with larger sample sizes about the effects of probiotics supplementation on birth weight are needed.

In addition, the dose or CFU of a probiotic is an important factor for the efficacy of probiotics supplementation on metabolic health in pregnant women. Among the ten RCTs, the dose or CFU of a probiotic is variable. Three studies with 10^10^ CFU probiotic counts, six studies with about 10^9^ CFU probiotic counts, and only one study with 10^7^ CFU probiotic counts were included and evaluated. Thus, it seemingly that the dose of more than 10^7^ CFU probiotic counts could show beneficial effects of probiotics supplementation on metabolic health in pregnant women. However, studies about specific doses of probiotics are limited, further studies about optimal dose or CFU of a probiotic supplementation in pregnant women are required. In addition, probiotic strains are also variable among the studies, and it is difficult to evaluate the effects of a specific probiotic species on metabolic health. Among the ten studies, Lactobacillus and Bifidobacterium were the mostly widely used strains. Thus, we speculated that Lactobacillus and Bifidobacterium may be beneficial probiotic strains for metabolic health in pregnant women. Currently, studies about the effects of specific probiotic strains are also limited. There is no consensus on the specific dose of probiotics and the ideal probiotic strains for the clinical intervention. Thus, further RCT studies that fully investigate and compare the efficacy among variable CFU doses and different probiotic strains are warranted, that are critically important to determine the optimal dose and ideal probiotic strains supplementation during pregnancy.

There are several limitations that should be considered: (1) the sample sizes were relatively small, ranging from 60 to 256 subjects. This might limit the power to estimate the effects of probiotics supplementation on pregnancy outcomes; (2) intervention durations of some studies are not clear, and several studies were with short duration, from diagnosis until delivery for 6–8 weeks; (3) the follow-up duration is short; (4) the stages of gestation are variable among healthy, obese pregnant women, and women with GDM, which can be a confounding factor for pooling the studies. Therefore, high-quality studies with longer intervention and larger sample size are needed.

To the best of our knowledge, this is the first systematic analysis to assess the effects of probiotics supplementation on metabolic health in the pregnant women, including healthy pregnant women, obese pregnant women and women with GDM. Despite the above limitations, this systematic analysis was more convincing than any previous single study. Indeed, our review has some notable strengths: (1) this review was strictly adhered to the PRISMA guidelines and PICO principle, and these methodologies can increase the robustness and validity of the results and conclusions; (2) only RCT studies were included in this review and all of them were objectively judged to be of high quality; (3) the parameters used to assess the clinical outcomes in all studies are strict and repeatable.

Next, the potential mechanisms underlying the beneficial effects of probiotics supplementation on pregnancy outcomes and metabolic health in pregnancy should be discussed [15]. Several potential points could explain the beneficial effects of probiotics during pregnancy. Pregnant women are susceptible to increased insulin resistance and glucose intolrance, and probiotics supplementation can improve glycemic control and insulin resistance. A systematic review with 17 RCTs showed that probiotic consumption significantly reduced FPG, fasting plasma insulin and HOMA-IR [38]. Oxidative stress has been shown to be present in hyperglycemia [39], and specific strains of lactic acid bacteria have been demonstrated to have antioxidant properties [40]. Moreover, probiotics can regulate the function of microbiota. Firstly, specific probiotics can balance the properties of aberrant indigenous microbiota [41]. Secondly, probiotics can ameliorate intestinal permeability [42]. It indicated that prebiotics improved gastric motility and gastric electrical activity in preterm newborns [43]. Thirdly, probiotics can regulate the secretion of proinflammatory cytokines [32]. It indicated that probiotics significantly decreased interleukin-6 (IL-6), tumor necrosis factor-a (TNF-a) and high sensitivity C reactive protein (hsCRP) levels. However, it is still unclear whether the impacts on the infants are due to the direct effects of probiotics through the milk or the placenta or adaptive responses to altered metabolism in mothers. Thus, further studies to elaborate the underlying mechanisms are urgently warranted.

## Conclusions

In summary, the prevalence of GDM and maternal obesity is increasing rapidly worldwide. Intervention during pregnancy is proven to be challenging, with limited efficacy and low compliance. Our study demonstrated that probiotics supplementation during pregnancy has beneficial effects on glucose metabolic health among pregnant women, including women with GDM, and even healthy pregnant women. More importantly, the safety and easy implementation of probiotics supplementation has been widely accepted. A better understanding of the role of probiotics supplementation can provide critical implications for the early prevention and treatment of abnormal metabolic status among pregnant women, and thus ensure a healthier future for the mothers, infants, and even throughout young adulthood. However, high-quality, large-scale clinical trials are urgently warranted to assess the optimal dose and ideal bacterial composition of probiotics, and long-term outcome of probiotics among pregnant women.

## Supporting information

S1 Checklist (DOC)
